# In-Hospital Mortality Risk Model of Gastric Cancer Surgery: Analysis of a Nationwide Institutional-Level Database With 94,277 Chinese Patients

**DOI:** 10.3389/fonc.2019.00846

**Published:** 2019-10-01

**Authors:** Zhouqiao Wu, Huimin Cheng, Fei Shan, Xiangji Ying, Rulin Miao, Jianhong Dong, Yihong Sun, Aman Xu, Yanbing Zhou, Yanong Wang, Lin Chen, Yingwei Xue, Hui Cao, Yawei Hua, Zekuan Xu, Minhua Zheng, Min Yan, Changming Huang, Jian Suo, Han Liang, Lin Fan, Jiankun Hu, Xiang Hu, Guoli Li, Peiwu Yu, Guoxin Li, Yiran Shi, Huayou Luo, Yong Li, Ming Xie, Tianxue Liu, Zhongyuan Zhang, Ting Shi, Ziyu Li, Jiafu Ji

**Affiliations:** ^1^Key Laboratory of Carcinogenesis and Translational Research (Ministry of Education), Department of Gastrointestinal Surgery, Peking University Cancer Hospital and Institute, Beijing, China; ^2^School of Statistics and Mathematics, Central University of Finance and Economics, Beijing, China; ^3^Center of Minimally Invasive Gastrointestinal Surgery, Shanxi Provincial Cancer Hospital, Taiyuan, China; ^4^Department of General Surgery, Fudan University Zhongshan Hospital, Shanghai, China; ^5^Department of General Surgery, The First Affiliated Hospital of Anhui Medical University, Hefei, China; ^6^Department of General Surgery, The Affiliated Hospital of Qingdao University, Qingdao, China; ^7^Department of Gastric Cancer and Soft Tissue Sarcomas, Fudan University Shanghai Cancer Hospital, Shanghai, China; ^8^Department of General Surgery, People's Liberation Army General Hospital, People's Liberation Army (PLA) Army General Hospital, Beijing, China; ^9^Department of General Surgery, Harbin Medical University Cancer Hospital, Harbin, China; ^10^Department of General Surgery, Ruijin Hospital, Shanghai Jiao Tong University, Shanghai, China; ^11^Department of General Surgery, Henan Cancer Hospital, Zhengzhou, China; ^12^Department of General Surgery, The First Affiliated Hospital of Nanjing Medical University, Nanjing, China; ^13^Department of Gastric Surgery, Fujian Medical University Union Hospital, Fuzhou, China; ^14^Department of Gastric Surgery, The First Hospital of Jilin University, Changchun, China; ^15^Department of Gastric Cancer, Tianjin Medical University Cancer Institute and Hospital, Tianjin, China; ^16^Department of General Surgery, The First Affiliated Hospital of Xi'an Jiaotong Hospital, Xi'an, China; ^17^Department of Gastrointestinal Surgery, West China Hospital, Chengdu, China; ^18^Department of General Surgery, The First Affiliated Hospital of Dalian Medical University, Dalian, China; ^19^Department of Gastrointestinal Surgery, Nanjing General Hospital, Nanjing, China; ^20^Department of General Surgery, The First Hospital Affiliated Hospital to Army Medical University (AMU) (Southwest Hospital), Chongqing, China; ^21^Department of General Surgery, Nanfang Hospital, Guangzhou, China; ^22^Department of General Surgery, Weifang People's Hospital, Weifang, China; ^23^Department of Gastrointestinal Surgery, First Affiliated Hospital of Kunming Medical University, Kunming, China; ^24^Department of General Surgery, Guangdong General Hospital, Guangzhou, China; ^25^Department of General Surgery, Affiliated Hospital of Zunyi Medical University, Zunyi, Guangzhou, China; ^26^Department of General Surgery, Penglai People's Hospital, Penglai, China; ^27^Centre for Global Health Research, Usher Institute of Population Health Sciences and Informatics, University of Edinburgh, Edinburgh, United Kingdom

**Keywords:** gastric cancer, surgical safety, mortality, national database, prediction model

## Abstract

**Background:** The objective of this study is to identify independent risks and protective factors and to construct a mortality prediction model for gastrectomy in the Chinese population.

**Study design:** This is a population-based prospective cohort at an institutional level. Seventy-two participating hospitals reported their annual gastrectomy data between 2014 and 2016, while 44 variables covering the institution and surgical information were included in the analysis. We used R software to encode and complete data pre-processing. The first difference model was applied to build the risk model. Data from 2014 and 2015 were assigned to risk model development, while data from 2016 was used for validation.

**Results:** In the included centers with 94,277 gastric cancer cases, the in-hospital mortality rate was 0.32%. The regression model revealed that provinces with low-middle GDP, hospitals with annual gastrectomy volume between 100 and 500, greater volume of urgent surgeries performed, larger proportion of males, and a higher proportion of liver metastasis were independent risk factors for mortality following gastric surgeries, while higher laparoscopic resection volume, greater volume of distal gastrectomy with B2 reconstruction, and larger proportion of palliative surgery were independent protective factors (*p* < 0.05, respectively). In the prediction test, the mean square error of the training set was 0.948, while that of the test set was 0.728, demonstrating the effectiveness of this model.

**Conclusions:** We constructed the first mortality risk prediction model for gastric cancer surgery in the Chinese population. The identified risk factors will help with the therapy selection, while further informing Chinese medical policy decision-makers.

## Introduction

Gastric cancer is still a common global malignancy with over one million new cases each year, where more than half occurs in China. It remains the second most commonly diagnosed cancer in the ethnic Chinese population (1). Although early stage cases can be treated with endoscopic therapy, the majority of cases are already locally advanced at the time of diagnosis, in which case, surgical resection is obligatory for the treatment. Despite the steady improvement of oncological survival in the recent decades, surgical complications after gastrectomy are still fairly common (18.3–36%) (2–4). Major complications including anastomotic leakage, peritonitis, bleeding, and ileus could significantly hinder postoperative recovery, or even threaten the patient's life if not properly managed (5). Mere delays in recovery also postpone adjuvant therapy, which may influence oncological survival, let alone intra-abdominal infectious complications, which are associated with earlier recurrence and reduced long-term survival rates (6, 7).

To reduce postoperative mortality and complications, the medical infrastructure has seen a rise in the number of national surgical databases, producing invaluable findings to inform both domestic and international medical communities (8–10). The China Gastrointestinal Cancer Surgical Union was founded in 2016 for the purpose of improving surgical quality and safety in China by documenting and sharing cases. As the first step, we collected the annual summary of 72 participating medical centers with nearly 200,000 gastric and colorectal patient data in China between 2014 and 2016, and created the China Gastrointestinal Surgery Database (CGSD). Using this database, we constructed the first mortality prediction model for gastrectomy in the Chinese population.

## Methods

### CGSD Database

The CGSD was established for risk factor stratification of gastric and colorectal cancer surgery in the Chinese population. All member institutions must submit its annual institutional summary data to the central database. The CGSD's final structure is expected to be similar to the ACS-NSQIP (American College of Surgeons—National Surgical Quality Improvement Program) and the Japanese NCD (National Clinical Database) programs (2, 9), which yield a collection of individual-level data in the form of patients' demographic characteristics, pre-existing comorbidities, pre-operative laboratory results, surgical details, and postoperative outcomes. The majority of the participating centers are tertiary hospitals, covering 88.2% (30/34) provinces of China, with 94,277 gastric and 90,076 colorectal cancer patients' data collected between 2014 and 2016.

One of the ultimate goals of the CGSD is to predict institutional mortality, we therefore developed a prediction model in this study. We assigned data from 2014 and 2015 to the risk model development (training set), while data from 2016 was used for prediction model validation (test set).

### Data Collection

Each participating institution reported their annual summary data of gastric cancer surgeries. In total, 44 variables were included in the analysis, including institution information (e.g., hospital name, hospital type, annual gastrectomy volume, number of surgeons, and beds in the team etc.), and surgical information (e.g., number of open and laparoscopic gastrectomy, number of total, distal and proximal gastrectomy, number of different reconstruction types, number of surgery after neoadjuvant therapy, number of palliative surgery etc.).

### Endpoints of the Study

In this study, we used in-hospital mortality as our primary endpoint. It was defined as death during the hospitalization of the surgical treatment, regardless of the length of hospital stay or cause of death. In addition, the other safety parameters including re-operation rate and post-operative stay were selected as the secondary endpoints.

### Data Analysis

We used R (Version 3.3.2) software to encode and complete data pre-processing automatically. For the missing data, we imputed them by applying K-nearest neighbor (KNN). The proportion of missing data were <10% of all included variables, and most variables (40/44) had <5% of missing data (Table S1). The 3-year average of variables of interest were described as mean (min. to max). If not indicated, the averages were weighted by center volume.

In the training set, we used the first difference model for the prediction model, because it could circumvent the issue of non-independence of repeated measurements in each center. This model has been frequently used for the purposes of trend prediction in repeated measurements (11). The model can be specified as:

$$\begin{array}{l} \Delta y=\sum_{i=1}^{k}\beta_{i}\Delta x_{i}+\Delta u, \end{array}$$

where Δ*y* and Δ*x*_*i*_ denote the first difference in response and predictors respectively. In other words, Δ*y*_*t*_ = *y*_*t*_ − *y*_*t* − 1_, Δ*x*_*it*_ = *x*_*it*_ − *x*_*it* − 1_, where *t* represents time, that is, different years of this study. β_*i*_ represents the influence of a unit of change in Δ*x*_*it*_ on Δ*y*_*t*_ (12, 13), and *e*_*t*_ denotes the residual. For example, β_*i*_ = 3 means that if *x*_*i*_ increases by 1 unit at time t, then *y* will increase by 3 units at time t.

In the section of prediction validation (test set), mean square error (MSE) is applied to evaluate the accuracy of the model prediction, which is calculated as below:

$$\begin{array}{l} \text{MSE}=\frac{1}{N}\sum_{i=1}^{N}{\left(Y_{i}-\hat{Y_{i}}\right)}^{2}, \end{array}$$

where *Y*_*i*_ and $\hat{Y_{i}}$ denotes the observed and predicted value, $(Y_{i}-\hat{Y_{i})}$ is known as residual. Lower MSE means better prediction accuracy. An MSE of zero, meaning that the estimators $\hat{Y_{i}}$ predicts observations *Y*_*i*_ with perfect accuracy.

All data analyses were conducted using R (version 3.3.2). A two-sided *P* value < 0.05 is considered as statistical significance. No ethical approval nor informed consent was required for this study under the domestic legislation.

## Results

### Demographic Characteristics

There are 72 hospitals reported the gastric cancer data to the database. Among them, 78% are general hospitals while the other 22% are cancer hospitals.

The average annual volume of gastric cancer surgery per institution was 585, which was conducted by an average of 9.6 surgeons per hospital. The average volume of open gastrectomy was 260.1 cases per year, while that of the laparoscopic ones was 135.2 cases per year. The average number of harvested lymph nodes was 27.4. A summary of the demographic characteristics is listed in Table 1.

**Table 1:** Summary of demographic characteristics.

**Parameter**	**Mean**	**Mean'**	**Min**	**Max**	**Preprocessing**
Provincial GDP level	–	–	–	–	Divided into 4 categories in decreasing order by the GDP of the hospital: 34.72, 33.33, 16.67, 15.28%
Hospital Type	–	–	–	–	Divided into: General hospital (78%), Cancer hospital (22%)
Male proportion	68.88%	68.97%	46.09%	81.41%	Number of Male patients/Number of all patients
Average Age	59.86	59.71	52.57	67.06	
Number of beds for gastric cancer surgery	78.76	80.77	15	328	
Number of gastric cancer surgeons	9.64	9.79	2	40	
Average surgeon per bed	0.14	0.14	0.03	0.67	Number of surgeons/number of beds
Annual gastrectomy volume level	–	–	–	–	Divided into 3 levels: <100(9.09%), 100–500(44.16%), >500 (46.75%)
Urgent surgery volume due to bleeding or obstruction	25.49	25.41	2	53	
Open gastrectomy volume	260.12	267.30	0^*^	1277	
Open total gastrectomy volume	113.02	115.7	0^*^	771	
Open distal gastrectomy volume	134.73	138.76	0^*^	495	
Open proximal gastrectomy volume	29.12	29.02	0^*^	345	
Laparoscopic resection volume	135.17	118.95	0^*^	614	
Laparoscopic total gastrectomy volume	52.66	46.30	0^*^	464	
Laparoscopic distal gastrectomy volume	77.44	68.45	0^*^	378	
Laparoscopic proximal gastrectomy volume	7.40	5.80	0^*^	84	
Number of average harvested lymph nodes	27.40	27.18	8	62	
Liver metastasis proportion	2.01%	2.06%	0%	21.69%	Number of liver metastasis/gastrectomy volume
Palliative surgery proportion	2.06%	1.87%	0%	12.39%	Palliative surgery volume/gastrectomy volume

*Mean' indicates the weighted average value of the training dataset (2014–2015)*.

* *Due to the fact that a few hospitals only conduct open gastrectomy or laparoscopic gastrectomy (with a few conversions), the number of 0 was reported, respectively*.

### Mortality

The in-hospital mortality rate was 0.3% (0–3.6%), and the other surgical safety outcomes are summarized in Table 2.

**Table 2:** Surgical safety and recovery outcomes.

**Outcomes (min. to max.)**	**Mean**	**Mean'**
Mortality, %	0.32(0–3.6)	0.35
Reoperation rate, %	1.46(0–7.08)	1.55
Postoperative stay, days	15.69 (7.50–26)	16.10

*Mean' indicates the weighted average value of the training dataset (2014–2015)*.

### Predictive Factors

The regression model revealed that low/middle GDP provinces, annual gastrectomy volume between 100 and 500, greater numbers of urgent surgeries performed due to bleeding or obstruction, higher reoperation rate, greater proportion of male patients, and a greater proportion of liver metastasis were independent risk factors for mortality after gastric surgery, while higher laparoscopic resection volume, higher distal gastrectomy with B2 reconstruction volume, and higher proportion of palliative surgery were the independent protective factors (*p* < 0.05 respectively, Table 3). All risks and protective factors listed above were measured at an institutional level.

**Table 3:** Risk model of in-hospital mortality.

**Variables**	**Status**	**Parameter estimation**	**95% CI**	***P*-value**
Provincial GDP level	middle-low GDP	0.97	0.37 to 1.57	0.002
Annual gastrectomy volume	100–500	0.75	0.10 to 1.40	0.002
Liver metastasis proportion	higher proportion	0.42	0.25 to 0.59	<0.001
Male patient proportion	higher proportion	0.23	0.08 to 0.37	0.004
Reoperation rate	higher rate	0.18	0.01 to 0.35	0.0380
Urgent surgery volume	Yes	0.17	0.02 to 0.33	0.027
Distal gastrectomy with B2 reconstruction volume	higher volume	−0.32	−0.54 to (−0.10)	0.005
Palliative surgery proportion	higher proportion	−0.32	−0.53 to (−0.12)	0.003
Laparoscopic resection volume	higher volume	−0.36	−0.69 to (−0.03)	0.037

### Prediction Model Evaluation

**Figure 1:**
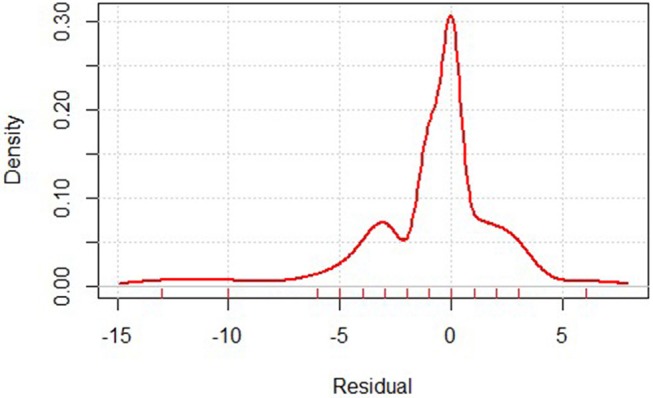
Residual distribution of gastric cancer mortality prediction model. Residual calculates the difference between the actual and predicted value.

The gastric mortality-risk prediction model shows that the MSE of the training set was 0.948, while that of the test set was 0.728. Most residuals in the model are concentrated near 0. The low MSE in the model shows the effectiveness of our predictive model (Figure 1).

## Discussion

This is the first report, based on the large-scale national surgical database that we have recently established, of institution-level risk stratification and in-hospital mortality prediction on gastric cancer surgeries in China.

The in-hospital mortality rate of gastric cancer surgery was 0.32% in our database, varying between 0 and 3.6% in different participating centers. This is comparable to the results from other Asian countries (0.3–1.2%) (3, 14), and seems lower than some studies from Europe or America (4.7–10%) (2, 8, 15). However, such difference cannot simply be explained by surgical quality or safety. One obvious reason is that the disease epidemiology (including severity) varied across regions, which substantially varied the surgical difficulties in different areas. Another possible explanation is that many other national databases also include emergency surgery with non-malignant causes, which may substantially increase the mortality [0.7 vs. 6.0% for right hemicolectomy (16)], while our database primarily focuses on cases of cancer. Although some urgent cases due to bleeding or obstruction were still performed (and those were indeed identified as risk factors in the results), the majority of the cases were elective procedures.

We chose in-hospital mortality rather than the 30-day mortality (a more frequently used parameter for surgical safety evaluation) as the primary endpoint in CGSD. This is because of the currently unsatisfactory follow-up system. In China, most gastric cancer patients are treated in tertiary hospitals in the cities (17), however many of the patients continue their follow-up treatment in the primary hospitals that are distant from the cities. In this case, it is difficult to obtain follow-up data updates, therefore, in-hospital mortality is a more practical and reliable parameter to evaluate the surgical safety in the current circumstances.

Many of our identified risk factors were also reported in the previous literature, such as urgent surgery (bleeding, obstruction) (4), and greater male proportion (3). In addition, our data supports the application of laparoscopy in gastric cancer patients, as the laparoscopic resection volume was identified as a protective factor. The advantages of the laparoscopic approach in colorectal surgery has now reached consensus among doctors, while its application in gastric cancer surgery remains in the early stage cases. The protective effect of the higher laparoscopic volume may be partly explained with the higher number of early stage cases in those centers, since the surgical procedures are standardized and face fewer technical difficulties. Similarly, the safety analysis of the CLASS-01 (Chinese Laparoscopic Gastrointestinal Surgery Study-01) trial has supported the use of laparoscopy in locally advanced gastric cancer cases (18), with similar oncological survival outcomes compared to the open procedures (19).

Higher liver metastasis proportion and higher palliative surgery proportion were identified as risk and protective factors, respectively, in our study. This is in agreement with the NCCN (National Comprehensive Cancer Network) and Japanese guidelines, which are, in general, against surgical therapy in M1 patients (20, 21). The surgical complexity and surgical trauma to multiple organs may explain the higher mortality in this type of patients. Yet, the Japanese guidelines also emphasize the possibility of hepatectomy for patients with a small number of metastatic nodules (20). A recent systematic review suggests better survival rates in favor of hepatectomy in this group of patients (22). With the recent advances in chemotherapy, more hepatectomies are performed for those gastric cancer patients with resectable nodules after conversion therapy. Our data address the short-term risks of this complex procedure, emphasizing the importance of safety evaluation when designing the surgical plan.

In addition to the aforementioned disease associated factors, our analysis also revealed many institutional factors which might influence mortality rates. Low GDP province and low annual volume seem to increase the in-hospital mortality of gastric cancer surgery. Similar results were also reported in the other literature (23, 24). One of the solutions for this is to relocate patients to select surgical theaters for complicated surgeries. Many European countries with low incidence of gastric cancer have applied this concept in practice by referring those patients to certain centers for gastrectomy (25). Greater volume often implies a greater level of technical competence from surgeons, which may result in enhanced safety outcomes (8, 24, 26). In addition, higher volume centers often indicate high-intensity intensive care units, the additional availability of multi-disciplinary teams and interventional radiology, effective prevention, and management of complications. These factors also influence the postoperative mortality (26). However, whether concentrating gastric cancer surgery fits the needs of the medical environment in China requires more research and discussion, given its large population and vast territories.

Distal gastrectomy was found to be a protective factor for mortality. This is in accordance with the literature reporting that distal gastrectomy is associated with fewer complications and thus lower in-hospital mortality when compared to the Japanese NCD data of distal and total gastrectomy (3, 4). Unfortunately, post-operative safety outcomes, i.e., complications, were not documented in a standardized manner in CGSD. It should be emphasized that morbidity remains substantial after gastrectomy. Our database also required complication rate data from each participant, but it varied between 0.3 and 33.1%. This is mainly because of the lack of standardized complication registration in China. Given that our union was devoted to this subject and has published the first consensus for standardized complication diagnosis and registration in China (27), several on-going prospective cohort studies are expected to reveal the complication rate and its severity after gastric cancer surgery in China. We believe these efforts would result in better data quality and thus better surgical outcomes in the future.

Considering the overall number of centers performing gastrectomy in China, our database has collected a portion of them and the majority are tertiary (3A-level) hospitals, which inevitably introduced selection bias in the current research. However, given the fact that most gastric cancer cases are treated in 3A-level hospitals in China (17), our findings may prove valuable to health care policy makers in China. Our prediction model reached a satisfactory result in predicting mortality, in which most residuals in the model are concentrated near 0, indicating nearly no difference between prediction and observation. This demonstrates its effectiveness. To reach a better sampling of the disease population, we are encouraging more hospitals to submit their data to the database. The number of participating centers continue to increase since its activation, and in 2018, CGSD has 85 participating members.

Another limitation to our study is that this institutional level database may not provide exclusive and conclusive answers to many detailed questions. Our analysis did not include any preoperative comorbidities or risk factors (e.g., weight loss or American Society of Anesthesiologists score), which could also influence surgical safety. The next step in data registration is to conduct individual level data collection which includes preoperative factors. The inclusion of such factors has begun in several centers in 2017, and the analysis of said individual data is on-going. It has been acknowledged that it is difficult to collect all details for a national database. Neither ACS-NSQIP nor the Japanese NCD included disease-specific data (e.g., staging and pathological outcomes) in their databases (8). This is mainly subject to the purpose of their databases: surgical safety. Moreover, maintaining such large-scale databases is challenging (10). Improving our Chinese gastrointestinal union database by learning from our international peers is necessary to ensure its quality and cost-effectiveness. As the first application, our analysis of the gross data still delivers many important insights into the clinical practice.

In conclusion, we have reported the first risk stratification study for gastric cancer surgeries, using a nationwide institutional level database. The surgical outcomes were satisfactory. These identified risk factors could be used in reference for future therapy selection, while serving as additional insight for Chinese health care policy makers.

## Data Availability

The datasets analyzed in this manuscript are not publicly available. Requests to access the datasets should be directed to ziyu_li@hsc.pku.edu.cn.

## Author Contributions

ZL, JJ, FS, and RM designed the study. The acquisition of data was conducted by ZW, HCh, FS, RM, JD, YSu, AX, YZ, YW, LC, YX, HCa, YH, ZX, MZ, MY, CH, JS, HLi, LF, JH, XH, GuolL, PY, GuoxL, YSh, HLu, YL, MX, TL, and ZL. The data analysis and interpretation was performed by HCh, ZW, XY, and ZZ. ZW, HCh, and TS drafted the manuscript. All authors extensively and critically revised the manuscript before submission.

### Conflict of Interest Statement

The authors declare that the research was conducted in the absence of any commercial or financial relationships that could be construed as a potential conflict of interest.
