# Supplementary material for: In-Hospital Mortality Risk Model of Gastric Cancer Surgery: Analysis of a Nationwide Institutional-Level Database With 94,277 Chinese Patients
Source: Front Oncol. 2019 Oct 1;9:846. doi: 10.3389/fonc.2019.00846 (PMC6779801; doi:10.3389/fonc.2019.00846)
Supplement: Supplementary file 1 [file Table_1.DOCX]

**Supplementary data**

Table S1. Data missing rate

| Variables | missing rate (%) |
| --- | --- |
| Year | 0 |
| Hospital | 0 |
| Province | 0 |
| Hospital type | 0 |
| Annual gastrectomy volume level | 0 |
| Average age | 0.65 |
| Number of beds for gastric cancer surgery | 0 |
| Number of gastric cancer surgeons | 0 |
| Routine abdominal exploration (Y/N) | 1.95 |
| Open gastrectomy volume | 1.3 |
| Thoracoabdominal approach volume | 3.9 |
| Open total gastrectomy volume | 1.3 |
| Open distal gastrectomy volume | 1.3 |
| Open proximal gastrectomy volume | 1.95 |
| Laparoscopic gastrectomy volume | 2.6 |
| Laparoscopic total gastrectomy volume | 9.09 |
| Laparoscopic distal gastrectomy volume | 9.09 |
| Laparoscopic proximal gastrectomy volume | 9.74 |
| Gastrectomy after neoadjuvant therapy volume | 1.95 |
| Number of average harvested lymph nodes | 2.6 |
| Liver metastasis volume | 0.65 |
| Ovarian metastasis volume | 2.6 |
| Peritoneal metastasis volume | 1.95 |
| Distal lymph node metastasis volume | 5.19 |
| Urgent operation volume | 2.6 |
| Palliative surgery volume | 4.55 |
| Gastrectomy after conversion therapy | 3.25 |
| Total gastrectomy with RY reconstruction volume | 1.95 |
| Total gastrectomy with other reconstruction volume | 0 |
| Distal gastrectomy with B1 reconstruction volume | 1.95 |
| Distal gastrectomy with B2 reconstruction volume | 1.95 |
| Distal gastrectomy with RY reconstruction volume | 4.55 |
| Distal gastrectomy with other reconstruction volume | 0 |
| Male proportion | 0 |
| Annual patient number per bed | 0 |
| Average doctor number per bed | 0 |
| Gastrectomy after neoadjuvant therapy volume | 3.9 |
| Liver metastasis proportion | 0.65 |
| Ovarian metastasis proportion | 2.6 |
| Urgent operation rate | 2.6 |
| Palliative operation rate | 4.55 |
| Gastrectomy after conversion therapy rate | 3.25 |
| Reoperation rate | 1.95 |
| Mortality | 1.3 |
